# The current issues and challenges in the management of floating knee injury: a retrospective study

**DOI:** 10.3389/fsurg.2023.1164032

**Published:** 2023-05-03

**Authors:** Guy Romeo Kenmegne, Chang Zou, Yixiang Lin, Yijie Yin, Shengbo Huang, Yue Fang

**Affiliations:** Trauma Center, West China Hospital of Sichuan University, Chengdu, China

**Keywords:** floating knee, polytrauma, femur fracture, tibia fracture, concomitant injury

## Abstract

**Purpose:**

The management of floating knee injuries is still controversial and challenging for trauma specialists. This study aims to evaluate the incidence of the floating knee in lower limb trauma, analyzing the challenges in its management, and factors affecting clinical outcomes.

**Methods:**

In this mono-center retrospective study, 36 consecutive patients were included. All individuals were diagnosed with an ipsilateral fracture of the femur and tibia, managed surgically according to their fracture pattern (Fraser classification), and the severity of the injury. The timing for each operation was determined based on the general condition of the patient and the local physiological condition of soft tissues. The patients’ clinical outcomes were finally evaluated based on their Karlstrom and Olerud scores and were categorized as excellent, good, acceptable, fair, or poor.

**Results:**

In this study, the mean follow-up period was 51.39 ± 16.02 months (11–130 months). Incidence of the floating knee was 2.32% in all lower limb traumas. From this number, 16 patients suffered from floating knee injury in the left lower extremity, and 18 in the right lower limb, while in 2 patients the condition was bilateral. The most common injury mechanism was road traffic accidents, accounting for 28 (77.78%) cases. The outcome was as follows; Excellent to good results in 22 (61.11%) cases, acceptable results in 2 (5.56%) cases, and fair to poor results in 12 (33.33%) cases according to the Karlström–Olerud scoring system. The most frequent early complications were wound infection and deep venous thrombosis in 5 (13.88%) of the cases. The most common late complication was common peroneal nerve palsy recorded in 2 (5.56%) cases.

**Conclusion:**

The presence of important concomitant injuries to the floating knee together with poor soft tissue conditions constituted important factors influencing possible management options and may have led to poorer clinical outcomes.

## 1. Introduction

Floating knee injury also known as “flail knee”, is usually classified under complex injuries of the lower limb (1, 2). The term was initially used in early 1975 by Blake and Mc Bryde to describe fractures of the ipsilateral femur and tibia (1). Although fracture to either femur or tibia is very common in trauma, the occurrence of both simultaneously in the same limb (ipsilateral) is unusual (1, 3). It is associated with high-energy trauma and is accompanied by other associated injuries as well as soft tissue lesions (ligaments and meniscus injury) (2, 4–6). There is limited data estimating the prevalence of this atypical injury among the population, however, there is a tendency with male predominance (3).

Fraser et al. (7). Initially classified floating knee injuries as; Type I: femur and tibia shaft fracture without knee joint articular surface involvement; Type II*:* fractures extended into the knee joint, Type II was further sub-divided into; Type IIa: tibial plateau involvement. Type IIb*:* intercondylar fracture of the distal femur, and Type IIc involving both the tibial plateau and the distal femur articular surface; This classification was recently modified by Ran to include disruption of the extensor apparatus; according to the author, Type I fractures are extra-articular fractures, Type II are intra-articular, and type III are associated patella fractures (8).

Floating knee injuries are frequently described in severely injured patients as open fractures with serious skin damage, with/without neurovascular injury. They present a life-threatening condition with mortality and limb amputation rates as high as 10% and 26% respectively (9–11).

The initial management plan is based on the overall clinical presentation of the patient and the local skin damage. Damage control surgery is usually applied.

While the peri-operative plan and the choice of fixation device remain challenging for orthopedic surgeons, the available literature recommends fixing the femur first to facilitate the management of the tibia. This plan allows for delay of tibia fixation in the medically unstable patient (10, 12, 13). The fixation method most recommended is double intramedullary nailing (7, 11).

Other authors have also proposed a treatment algorithm for floating knees based on patient/limb condition, and Fraser’s classification system. However, it is prudent to adapt the treatment plan to the skin, soft tissue damage, joint articular surface involvement, and the degree of comminution of the fracture fragment which represents the best postoperative functional prognosis and also best minimizes complications (3, 9, 14) including infection, nonunion, mal-union, and joint stiffness (15) each of which may lead to functional impairment and unsatisfactory results.

This article will discuss incidence of floating knee injuries in lower limb trauma, the challenges in its management, and factors affecting the clinical outcome.

## 2. Patients and methodology

This retrospective descriptive study encloses patients admitted with a diagnosis of ipsilateral fracture of the femur and tibia, managed in a single first-level trauma center between January 1st, 2011 and December 31st, 2021.

Patients presenting with an ipsilateral fracture of the femur and tibia were identified among a set of 1976 patients who sustained a fracture in the lower limb. All patients were treated by a team of highly specialized orthopedic trauma surgeons some of whom co-authored this study.

Given the nature (poly-trauma) of injury, the severity was evaluated using the injury severity score (ISS), grading the lethality of the injury from 0 to 75.

As a retrospective study, the ethical committee approval was obtained from the institutional review board of our institution, and informed consent was obtained from all patients after explaining all details regarding the treatment protocol.

**Inclusion criteria**:

All patients with floating knee injury; all patients with lower limb trauma, irrespective to the mechanism and the nature of the injury; Patients with skeletal maturity (defined as those aged 15 years and above); patients who were alive on presentation; and patients managed surgically with a minimum follow-up period of 12 months.

**Exclusion criteria:**

Patients with any history of malignancy or Pathologic fractures, Patients whose follow-up was discontinued, Patients with insufficiency fractures (fractures occurring without trauma), Patients with a history of metabolic or other types of bone diseases, and Patients with periprosthetic fractures.

A total of 46 patients were identified with floating knee; however, only 36 patients met the inclusion criteria and were selected for the final review. All 36 patients included in the study had a good and complete follow-up period.

### 2.1. Perioperative management

All admitted patients were initially stabilized in the emergency unit. In cases of polytrauma, appropriate management was given with respect to the Advanced Trauma Life Support (ATLS) protocol. All patients were evaluated to exclude neurovascular and additional injuries. The initial injury stabilization was applied according to the patient’s specific and general physiological condition.

Patients with open injuries classified according to the Gustilo-Anderson classification system were scheduled for emergency debridement, sterile coverage, and external fixation of the fracture in the context of damage control surgery/orthopedics (DCS or DCS).

Patients presenting with tidy wounds received a first-generation cephalosporin (1 g cefazolin was the first choice, and was discontinued 24 h post-operatively for Gustilo I and II open wounds) while those with untidy wounds received additional aminoglycoside (gentamycin 240 mg) and metronidazole 500 mg. This was continued for 72 h for Gustilo type III open fractures. Prophylactic tetanus therapy was also administered to all those who presented with open injuries.

In patients with severe soft tissue defect, in order to speed the soft tissue recovery, we used combined antibiotic impregnated bone cement with VAC (vacuum-assisted closure “VAC”); Following thorough debridement, a prepared pie-shaped antibiotic-laden bone cement was used to fill the soft tissue cavity; the wound was then veiled by the tailored VAC sponge, sutured with surrounding normal skin to ensure that the entire wound surface is covered; and a semipermeable was used to seal the wound and the VAC dressing. The VAC dressing was regularly changed every seven to ten days until a clean, red, and granulating wound bed was attained. The following antibiotic-laden bone cements were routinely used: gentamicin bone cement (1.6% gentamicin), vancomycin bone cement (15% vancomycin), and cefoperazone bone cement (10% cefoperazone). The use of VAC in this approach allows for complete suction of seepage, necrotic tissues, and pus from the wound area using a negative-pressure device; the antibiotic-impregnated cement is useful in infection prevention and improving flesh regeneration; and this method allows for faster soft tissue recovery and the possibility of early skin graft.

All patients received deep venous thrombosis (DVT) prophylaxis with low molecular weight heparins (LMWH) adjusted according to the patient’s weight (0.2–0.4 ml). Routine white blood cell counts (WBC), C-reactive protein (CRP), and erythrocyte sedimentation rate (ESR) were regularly checked to exclude any case of wound infection or sepsis.

Those in unstable general condition and with closed fractures were either admitted to the trauma ward, or the surgical intensive care unit (SICU) for intensive management. Patients with suspected haemothorax or pneumothorax, had chest drains.

Patients presenting with polytrauma were subjected to full body CT scan examination, as per Trauma protocol, with 3D reconstruction where needed. As a result, all patients with fluctuating or altered consciousness levels and suspected of having Traumatic Brain Injury (TBI) evidenced by a CT scan, were systematically referred to the neurosurgery unit for further management. The fracture fixation was postponed until the patients were stabilized and scheduled for staged treatment. Early definitive fixation (early total care/ETC) was preferred for the less severely injured patients.

The perioperative planning was based on standard radiographic images and 3D CT scans. The radiographic and CT scan images were used for further classification of fracture and surgical planning. Venous duplex ultrasound was done before the procedure to exclude DVT in every patient.

All patients who were eligible for surgical stabilization received general or spinal anesthesia and a pre-operative (30 min before induction of anesthesia) administration of prophylactic antibiotic in addition to, 1–2 g of intravenous tranexamic acid to minimize intraoperative blood loss. A tourniquet was used in selected cases when necessary.

For the surgical approach, the reduction technique and the implant selection was decided based on the fracture pattern, and its classification according to Fraser’s classification (7), the patient’s physiological state at presentation, and the condition of soft tissue. All operations were therefore carried out when the patients were hemodynamically stable.

Preferably, Antegrade Intramedullary Nails and plates were used for the fixation of femur fractures, while Intramedullary nailing approach and plates were used for tibial fractures. In cases of severe soft tissue compromise, external fixation was preferred for definitive fixation. Accompanying ipsilateral patella fractures were operated on using the tension band technique.

After fracture fixation, the Lachman’s and posterior drawer’s tests were done, intraoperatively under anesthesia, to exclude cases of anterior and posterior cruciate ligament injury, which when suspected was referred to a sports medicine specialist for appropriate ligament reconstruction surgery.

The postoperative protocol consisted of temporary immobilization, infection prophylaxis, and proper wound dressing that ensured proper wound and soft tissue healing. Patients with serious soft tissue defects received skin grafts (patient number six in Table 1).

**Table 1:** Patients’ demographics.

Case	Age	Gender	ISS	MOI	SOI	Fraser	OA/OTA		Gustilo	Concomitant injuries	Management plan		Complications	Karlström–Olerud score	LOS
							Fe	Ti			Fe	Ti			
1	57	M	16	RTA	R	IIB	33C2	42C3	Ti: I	- Ipsilateral talus fracture - Ipsilateral 1st metatarsal fracture - Common peroneal nerve lesion	Plate	IM		Good	25
2	30	F	22	RTA	R	IIB	33C2	42C3	Fe and Ti IIIA	- Contralateral olecranon fracture - Ipsilateral ankle fracture - Tile A pelvic fracture	Plate	IM		Excellent	17
3	45	M	16	Heavy object	L	I	32C2	42C2	Fe:II	- Ipsilateral patella Fracture - Ipsilateral first metacarpal fracture - Ipsilateral popliteal artery injury - Ipsilateral tibial nerve	Antegrade IM	IM		Excellent	32
4	25	M	41	RTA	B	R:IIA L:IIB	R: 33C2; L: 33B1	R:42C3; L:42B2	L(Ti:IIIc)	- TBI - Intra-abdominal organ(liver, spleen) injury - Unilateral Multiple rib fracture - Lung contusion	- L: above-knee amputation - R: antegrade IM	R: plate		Good	15
5	31	M	27	RTA	R	I	32B2	41A3		- Ipsilateral radius - Bilateral clavicle - Vertebral body T4,5,7,8and 12 + L1 fracture(Frankel D) - Bilateral multiple ribs fracture - TBI	Antegrade IM	IM		Excellent	36
6	43	M	25	RTA	R	I	32B3	42B3	Ti: IIIA	- Unilateral multiple rib fracture - Lung contusion - Superficial peroneal nerve lesion	Antegrade IM	External fixation	- Infection - Soft tissue defect(calf muscle flap)	Acceptable	47
7	40	M	25	RTA	L	I	32B2	41A3	Fe:I Ti:II	- L2-3 vertebral body fracture, - Ipsilateral lateral malleolus fracture, - Fracture of contralateral tibia	External fixation: Illizarov	External fixation		Fair	24
8	25	M	25	Fall from height	L	I	32A2	42B2	Fe: II Ti: IIIA	- Ipsilateral lateral cuneiform avulsion fracture - T12,L1 vertebral body compression fracture	Antegrade IM	External fixation	Infection	Fair	10
9	22	M	43	RTA	L	IIB	33C2	42C3	Fe: II Ti: IIIB	- Contralateral acetabular fracture, - Pelvic (pubic ramus) fracture, - Ipsilateral medial malleolus fracture, - Ipsilateral 2nd metatarsal fracture - Unilateral Multiple ribs fracture - Lung contusion - Ipsilateral popliteal artery - Ipsilateral common peroneal nerve injury	Plate	IM	Common peroneal nerve palsy	Fair	21
10	69	M	25	RTA	L	IIB	33C2	42C3	Fe: II Ti: IIIB	- Ipsilateral open lateral malleolus fracture Gustilo IIIB, - Ipsilateral open fracture of distal phalanges of the 1st toe (Gustilo II) - Ipsilateral radius fracture, - Ipsilateral patella fracture, - T12 vertebral body fracture	Plate	IM nailing	Tibia nonunion	Fair	19
11	46	M	16	RTA	L	IIA	33C2	42C2	Fe: II	- Ipsilateral patella fracture	Antegrade nailing	Plate		Excellent	28
12	49	M	25	Heavy object	R	IIC	33C3	41C2	Fe: IIIA, Ti: IIIB	Bilateral malleolar fracture	Plate	Plate	Knee stiffness	Fair	41
13	70	F	34	RTA	L	IIC	33C3	41C2	Fe: II Ti: II	- Contralateral humerus - Fracture of the contralateral middle phalange, - Intraabdominal hemorrhage - Unilateral multiple ribs fracture - Lung contusion	Plate	Plate	- Wound Infection, - Femur nonunion	Fair	17
14	60	M	34	RTA	R	IIC	33C3	41C2	Fe: IIIB, Ti: IIIA	- TBI, - Contralateral single rib fracture, - Contralateral clavicle fracture, - Contralateral femoral shaft fracture	External fixation	External fixation		Good	26
15	46	F	29	RTA	L	I	32A2	42A1	Fe: I Ti: I	- Tile C2 Pelvic fracture, - Pelvic organ (bladder) contusion - Contralateral medial malleolar open fracture (Gustilo IIIB)	Antegrade IM	Anterograde IM		Excellent	20
16	35	M	16	RTA	R	IIA	32B3	41C2		Posterior tibial artery injury	Antegrade IM	Plate		Excellent	13
17	42	M	34	Fall from height	B	L: I, R: IIA	Fe: L(32C3); R(32B2)	Ti:L(42C3); R(42C2)		- Left Pilon fracture - Bilateral lower extremity crush injury, - Right calcaneus fracture, - Unilateral multiple rib fracture, - -Fracture of left metatarsal bone	- L: antegrade IM, - R: antegrade IM	- L: IM, - R: plate	Infection - Left Femur nonunion	Poor	23
18	30	F	16	RTA	R	I	32A2	42B3	Fe: I Ti: II	- Ipsilateral navicular avulsion fracture - Ipsilateral achilles tendon disruption	Antegrade IM	IM		Excellent	12
19	29	M	25	RTA	L	I	32A2	42C3	Fe: I Ti: I	- Tile B pelvic fracture, - Bladder injury	Antegrade IM	IM		excellent	13
20	49	F	16	Fall from height	R	I	32A2	42B3	Fe: I T:II	- Contralateral wrist dislocation, - Ipsilateral popliteal artery injury	Antegrade IM	External fixation	Ischemic necrosis of the calf	Fair	11
21	44	M	16	RTA	R	IIB	33C2	42C3	Ti: IIIB	- Femoral artery injury - Ipsilateral tibial nerve injury - Ipsilateral common peroneal nerve injury	Plate	External fixation	Common peroneal nerve palsy	Fair	12
22	32	M	25	RTA	R	IIB	33B1	42C3	Ti: II	- Ipsilateral medial maleollus fracture, - Ipsilateral radius fracture - Ipsilateral 1st and 3rd metatarsal fracture - TBI	Plate	External fixation		Good	15
23	57	M	29	Fall from height	R	IIB	33C3	42C2	Fe: II	- Contralateral femur fracture, - Tile A pelvic fracture, - Ipsilateral acetabular fracture - Ipsilateral Sciatic nerve contusion	Plate	Plate		Acceptable	19
24	31	M	20	RTA	R	IIC	33C2	41C2	Fe: II	- Bilateral multiple Ribs fracture - Bilateral lung contusion - Ipsilateral clavicle fracture	External fixation	External fixation		Good	21
25	25	M	34	RTA	L	IIA	32C2	41C1		- Ipsilateral patella fracture, - Contralateral tibial plateau fracture, - Bilateral kidney, liver, spleen and bilateral adrenal gland contusion, - Bilateral lower extremities compartment syndrome, - Ipsilateral multiple ribs fracture,	Plate	Plate	- DVT of the right common femoral vein, - Bilateral calf intermuscular DVT	Poor	48
26	56	F	16	RTA	R	IIA	32A1	41C1	Fe: I	- Bilateral multiple ribs fracture	Antegrade IM	Plate	- DVT (popliteal vein)	Excellent	30
27	55	F	9	RTA	R	IIC	33C2	41C2	Fe: II		Plate	Plate		Excellent	19
28	40	M	37	RTA	L	IIC	33C3	41C1		- Ipsilateral patellar fracture, - Contralateral femoral condyle fracture, - Multiple maxillofacial fracture, - Ipsilateral eyeball trauma, - Fracture of vertebral body T1T4, - TBI	Plate	Plate	- DVT (small saphenous vein)	Good	23
29	30	M	16	RTA	R	I	32A3	42A1	Ti: II	- Unilateral multiple ribs fractures,	Antegrade IM	IM		Excellent	26
30	64	M	16	Fall from height	R	IIA	32B3	41C2	Fe: I Ti: II	- Ipsilateral patella fracture, - Contralateral pilon fracture	Antegrade IM	Plate		Excellent	12
31	24	M	25	Fall from height	R	I	32C2	42A3	Ti: I	- Ipsilateral patella and talus fracture, - Contralateral fracture of the 4th metatarsal and 5th phalanx, - Bilateral lateral malleolar fracture, - Unilateral multiple ribs fracture with ipsilateral lung contusion.	Antegrade IM	IM		Good	21
32	48	F	16	RTA	L	IIA	32C2	41C1		- Contralateral calcaneus fracture - Contralateral clavicle fracture	Antegrade IM and plate	plate		Good	19
33	59	M	38	RTA	L	IIB	33C1	42A2	Ti: I	- Ipsilateral sciatic nerve injury, - Ipsilateral common peroneal nerve injury - Ipsilateral tibial nerve injury, - TBI - Contralateral parietal bone fracture - Unilateral single rib fracture	Plate	IM	- Sciatic nerve palsy	Poor	26
34	65	M	27	RTA	L	IIC	33C3	41C3	Fe: II Ti: I	- Tile C pelvic fracture - Ipsilateral calcaneus fracture - Bilateral multiple ribs fracture - Bilateral pulmonary contusion	Eternal fixation	Eternal fixation	- Wound infection - Septic shock - Deep popliteal vein DVT)	Fair	23
35	29	M	34	RTA	L	I	32B2	42A1	Ti: IIIA	- Ipsilateral patella fracture, - Contralateral femur fracture, - Ipsilateral wrist hamate fracture, - TBI - Frontal bone fracture,	Plate	IM		Good	15
36	67	M	25	RTA	L	IIB	33C3	42C3		- Ipsilateral patella fracture - TBI	Plate	Plate	Deep popliteal vein DVT)	Excellent	16

MOI, mechanism of injury; ISS, injury severity score; SOI, side of injury; Fe, Femur; Ti, Tibia; LOS, length of stay; IM, intramedullary nailing; RTA, route traffic accident; DVT, deep venous thrombosis; L, left; R, right; B, bilateral.

Rehabilitation (physiotherapy) was initiated as soon as proper wound healing was achieved (usually ten to 21 days following surgery); patients were allowed full range of motion (ROM) of both hip and knee joints (with isometric for quadriceps & isotonic for hamstrings, as tolerated). Non-weight-bearing ambulation using crutches was permitted for six weeks (if the contralateral limb was not affected), followed by partial weight-bearing. Full weight-bearing was allowed only after clinical and radiological union had been con­firmed. Active ROM exercises were delayed in some patients who had knee-spanning external fixation.

On average, patients achieved full weight bearing after four weeks for extra-articular fractures and eight to ten weeks for intraarticular fractures.

The discharge of patients from the hospital was generally dictated by the patient’s general clinical condition, while the hospital length of stay (LOS) was determined by the severity of the initial trauma.

On discharge, a postoperative follow-up in the outpatient orthopedic clinic was scheduled at two weeks, one month, and once every three months for one year, then yearly thereafter.

The routine follow-up consisted of radiographic control (anteroposterior, lateral, and oblique) of the injured segments, evaluation of functional outcome (using the Karlstrom and Olerud grading system) (16), and complications. At discharge, oral rivaroxaban and Loxoprofen sodium were prescribed at a dosage of 10 mg/day and 180 mg/day (Q8h) respectively for a period of three weeks following discharge to prevent venous thrombosis embolism (VTE) and pain on a later stage.

Postoperative complications documented were classified as early; Wound infection, DVT, ischemic necrosis, and septic shock, and late*;* knee stiffness, nonunion, and nerve palsy*.* All clinical and functional outcomes were documented in patient files at 12 months of follow-up.

### 2.2. Data collection and statistical analysis

Patients’ data were recovered from the hospital information system and included in a database created on Excel 2010 program by Microsoft (Microsoft Corporation, Redmond, Washington, USA) for further processing. This data included demographic variables (age, sex, mechanism, and side of injury), and clinical data [injury severity score (ISS), fracture type, concomitant injury, management protocols, and hospital length of stay]. The final general conditions of the patients and their functional outcomes were obtained via telephone interviews at the final review ahead of the writing of this article. Descriptive statistics were performed to summarize the characteristics of the study group and subgroups. Continuous variables were expressed as mean ± standard deviation (SD). Categorical variables were displayed as counts (*n*) and percentages (%). All statistical analyses were done using SPSS version 20.0 (SPSS, IBM, USA).

## 3. Results

This study screened a total of 1,976 patients with lower limb trauma and identified a total of 46 cases of the floating knee. Finally, 36 patients, 28 males, and 8 females, with 38 floating knees participated in this study. Ages varied between 22 and 70 years (mean 43.58 ± 14.56). The injury severity score (ISS) varied between 9 and 43 (mean 24.80 ± 8.48). The hospital length of stay (LOS) was from 10 to 48 days (22.08 ± 9.44); the mean follow-up period was 51.39 ± 16.02 months (in 11–130 months).

Of the patients, 16 suffered from fractures on the left lower extremity, 18 suffered from a fracture in the right lower limb, and 2 patients suffered from bilateral lower limb fractures in the context of ipsilateral femur-tibia fractures (as there was some other existing concomitant fracture). Overall, 28 (77.78%) of our cases sustained a road traffic accident, 6 (16.67%) cases had a history of a fall from an altitude, and 2 (5.56%) were injured by heavy objects.

Among the femoral fractures, 16 were fixed only with intramedullary nails and 1 case with combined intramedullary nails with plate fixation. 16 patients underwent fixation with plate exclusively. External fixators were used as permanent fixation in 4 fractures.

Of the tibial fractures, 13 were fixed with an intramedullary nail, 14 with a plate, and 1 patient managed with a combined intramedullary nail and plate. External fixators were used as permanent fixations in 9 cases. There was 1 (2.78%) case of above-knee amputation.

In this study, 35 cases presented with concomitant injuries and are enumerated as follows; 14 cases of neurovascular injuries (2 sciatic nerve injuries, 4 common peroneal nerve injuries, 3 tibial nerve injuries, 1 superficial peroneal nerve injury, 1 femoral artery injury, 3 popliteal artery injuries, and 1 posterior tibial artery injury.); 8 cases of TBI, 2 of which presented with skull fractures; 9 cases of ipsilateral patella fractures; 14 cases of traumatic chest injury including 14 cases of rib fracture and 7 of lung contusion; 6 cases of pelvis fractures including 2 acetabular fractures (1 case with associated sciatic nerve contusion); 2 cases of bladder contusion; 1 case of bilateral kidney and adrenal gland contusion; 3 cases of traumatic injury to the abdomen with 1 case of intraabdominal hemorrhage and 2 cases of intra-abdominal organ (liver and spleen) injury.

Also mentioned were several cases of associated appendicular skeletal fractures with a single case of bilateral lower limb compartment syndrome.

In our series, fractures were open in 23 (60.52%) tibial fractures and in 21 (55.26%) femoral fractures (with Gustilo type II being the most encountered in both cases: 19.44% and 27.78% respectively). The most frequent early complications were wound infection and deep venous thrombosis, while the most encountered late complication was common peroneal nerve palsy in 2 (5.56%) cases (Table 1).

The fracture classification was performed according to the OA/OTA and Fraser classification system and summarized as shown in Table 2. According to these systems, a majority (34.21%) was classified as type I under the Fraser classification, then type 33C2/33C3 (femur = 21.05%) and 42C3 (tibia = 23.68%) under AO/OTA classification.

**Table 2:** Distribution of patients by AO/OTA and Fraser classification.

AO/OTA classification				Fraser classification	
Femur		Tibia		Types	Number of cases *n* (%)
Types	Number of cases *n* (%)	Types	Number of cases *n* (%)		
33C2	8 (21.05)	42C3	9 (23.68)	I	13 (34.21)
33C3	8 (21.05)	41C2	6 (15.78)	IIA	8 (21.05)
32A2	5 (13.15)	42C2	5 (13.15)	IIB	10 (26.31)
32B2	4 (10.52)	41C1	4 (10.52)	IIC	7 (18.42)
32C2	4 (10.52)	42B3	3 (7.89)		
32B3	2 (5.26)	42A1	33 (7.89)		
33B1	2 (5.26)	41A3	2 (5.26)		
32B3	1 (2.63)	42B2	2 (5.26)		
32C3	1 (2.63)	42C3	1 (2.63)		
32A1	1 (2.63)	41C2	1 (2.63)		
32A3	1 (2.63)	42A3	1 (2.63)		
33C1	1(2.63)	41C3	1(2.63)		

The result of the follow-up revealed excellent to good results in 22 (61.11%) cases, acceptable results in 2 (5.56%) cases, and fair to poor results in 12 (33.33%) cases according to the Karlström–Olerud scoring system at one-year of follow-up.

## 4. Discussion

The floating knee is a rare injury, and its incidence is not revealed in literature (5). This study is significantly beneficial in that, it is the only study with a follow-up period of up to 10 years, presenting the incidence as well as the clinical outcomes.

From the initial pool of 1,976 patients with lower limb trauma, the incidence of the floating knee was evaluated at 2.32% putting the incidence at 0.23% per year. This incidence is however expected to increase in the coming decade, with the trend of population expansion, illiteracy, limitations of modern infrastructures in most developing countries, and the recrudescence of high-energy trauma such as road traffic injuries and falls from heights.

In a study conducted by Dwyer et al. (17), it was observed that the male gender (54 men vs. six women), younger age (average 26.8 years), and road traffic accidents (57 out of 60 cases) were the most preponderant factors in floating knee injuries. These findings were widely discussed by several similar works in literature (7, 12, 18–21).

Almost all floating knees in this study were secondary to high-energy trauma, in men (28 males: 8 females) and ages ranging between 22 and 69 years. The findings in the study were consistent with those reported by previous authors.

Piétu et al. (22) in a retrospective multi-centric observational study, reviewed 172 floating knee injuries; according to Fraser's classification, 71.5% of the cases were type I, 8.2% were type IIA, type IIB was present in 11.6%, while type IIC present in 8.7%; one out of 69.2% of the patients had an open fracture. The average ISS was 19.5 and there were 37.7% cases of poly-traumatized (ISS > 18). Intramedullary nailing (IM) was the gold standard (73% of femoral fractures, and 54.4% of tibial fractures) while 25% of patients benefited from external fixation.

In the current study, according to Fraser's classification, 34.21% of the cases were type I, 21.05% were type IIa, 26.31% were type IIb, while type IIc was present in 18.42% of cases. Additionally, open fractures were present in 80.56% of all patients.

The mean ISS was 24.80 ± 8.48; a value slightly higher than that reported by Piétu et al. (22). However, this remains lower than the report from another scholar who reported a mean ISS as high as 39.05 (3). This could simply reflect the severity of the floating knee with associated injuries in a context where disregard for road safety, non-compliance with the Highway Code, and speeding are common.

The management approach of floating knee remains controversial among trauma surgeons; most authors supported that, an aggressive approach with early fixation of both femur and tibia and mobilization improves the clinical results (8, 13, 15, 21, 23, 24); in the other hand, some scholars preferred ETC (early total care) only for cases of stable patients and DCO (damage control orthopedics) in unstable or in extremis patients (25). However, a large debate between ETC and DCO for patients in borderline status remains. In our opinion, in uncertain cases, DCO is ideal considering the potential reduction in surgical time, bleeding risk and risk of metabolic shock response. This idea was previously supported in the current literature (26). Moreover, the principle of ETC is usually limited to patients with good skin and soft tissue conditions and a staged treatment protocol with temporary external fixation (DCO) is needed in those presenting poorer conditions; we therefore considered the principles of orthopedic damage control as the safest solution initially in the later conditions (patients with poorer condition). With this protocol, one important complication (knee joint stiffness has to be avoided); in this case we always recommend the conversion of external fixator into internal fixation as soon as the conditions permit and early transfer of patients to rehabilitation department.

The choice of implants were highly dependent on the fracture pattern or type, with/without articular surface involvement, soft tissue and skin condition, available resources, surgical capability, and both surgeon and patient preference (14, 15). Additionally, the impact of the osteosynthesis technique on the overall physiology of the patient should be kept in mind (27, 28).

**Figure 1:**
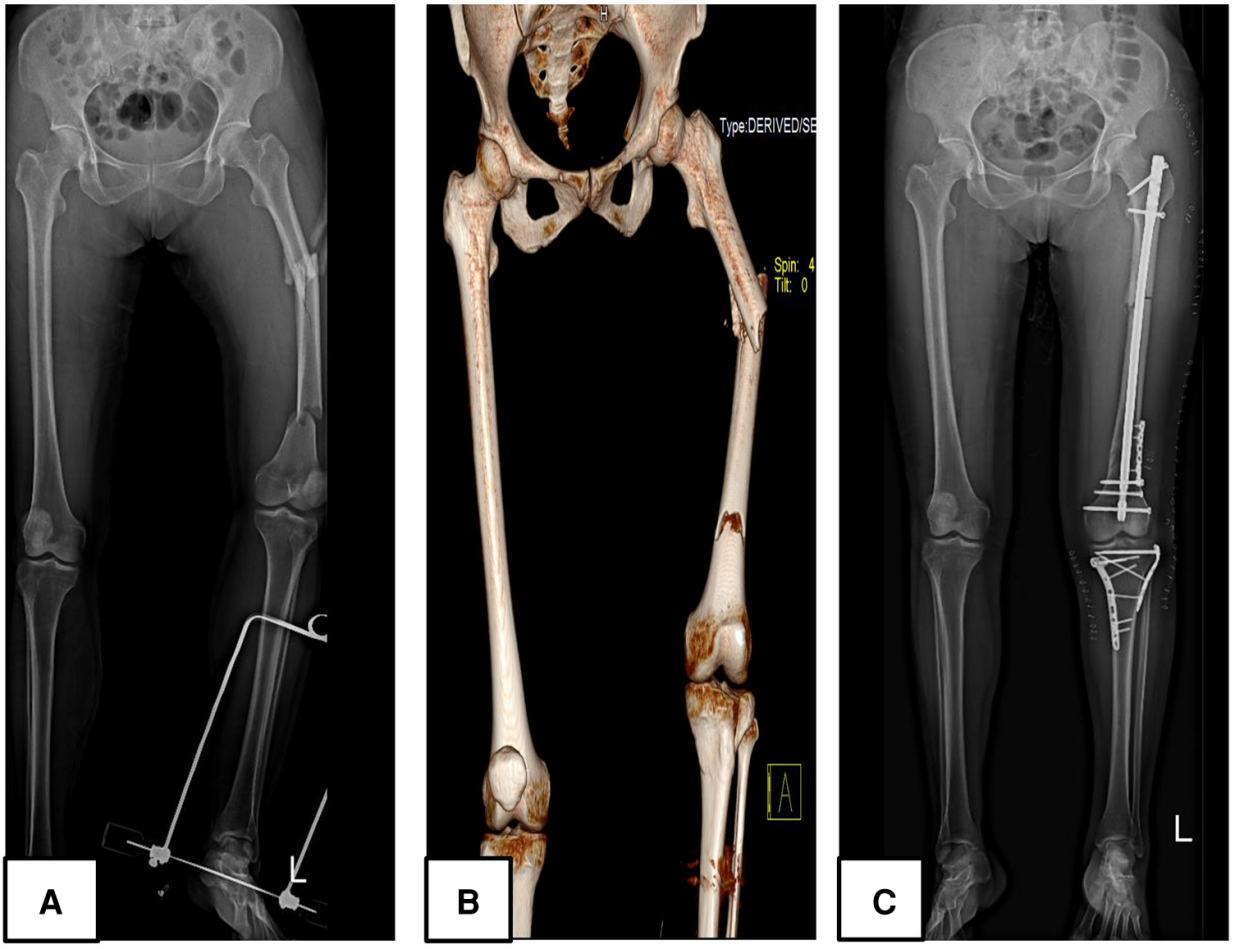
A 48 years-old female patient (patient number 32) who sustained a Fraser type IIA floating knee following a road traffic accident managed surgically; (**A,B**) represent preoperative plain radiograph and 3D reconstruction CT images respectively; (**C**) displays a postoperative plain radiograph of the lower limb, the femur was managed with an intramedullary nail combined with a reconstruction plate and the tibia was treated with a dual plate.

**Figure 2:**
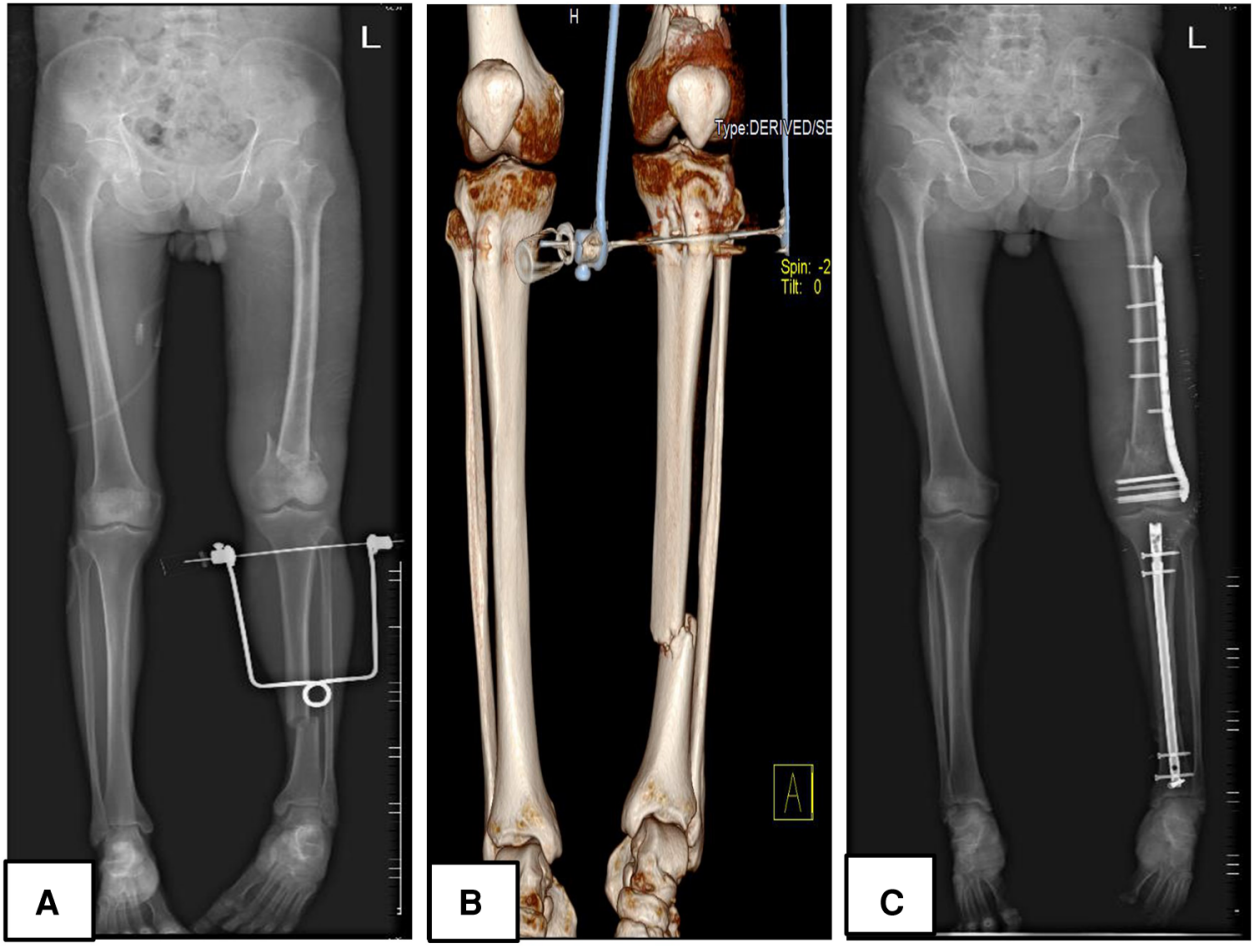
A 59 years-old male patient (patient number 33) who sustained a Fraser type IIB floating knee following a road traffic accident managed surgically; (**A,B**) represent preoperative plain radiograph and 3D reconstruction CT images respectively; (**C**) displays a postoperative plain radiograph of the lower limb, the femur was managed with a plate while the tibia was treated with an intramedullary nail.

**Figure 3:**
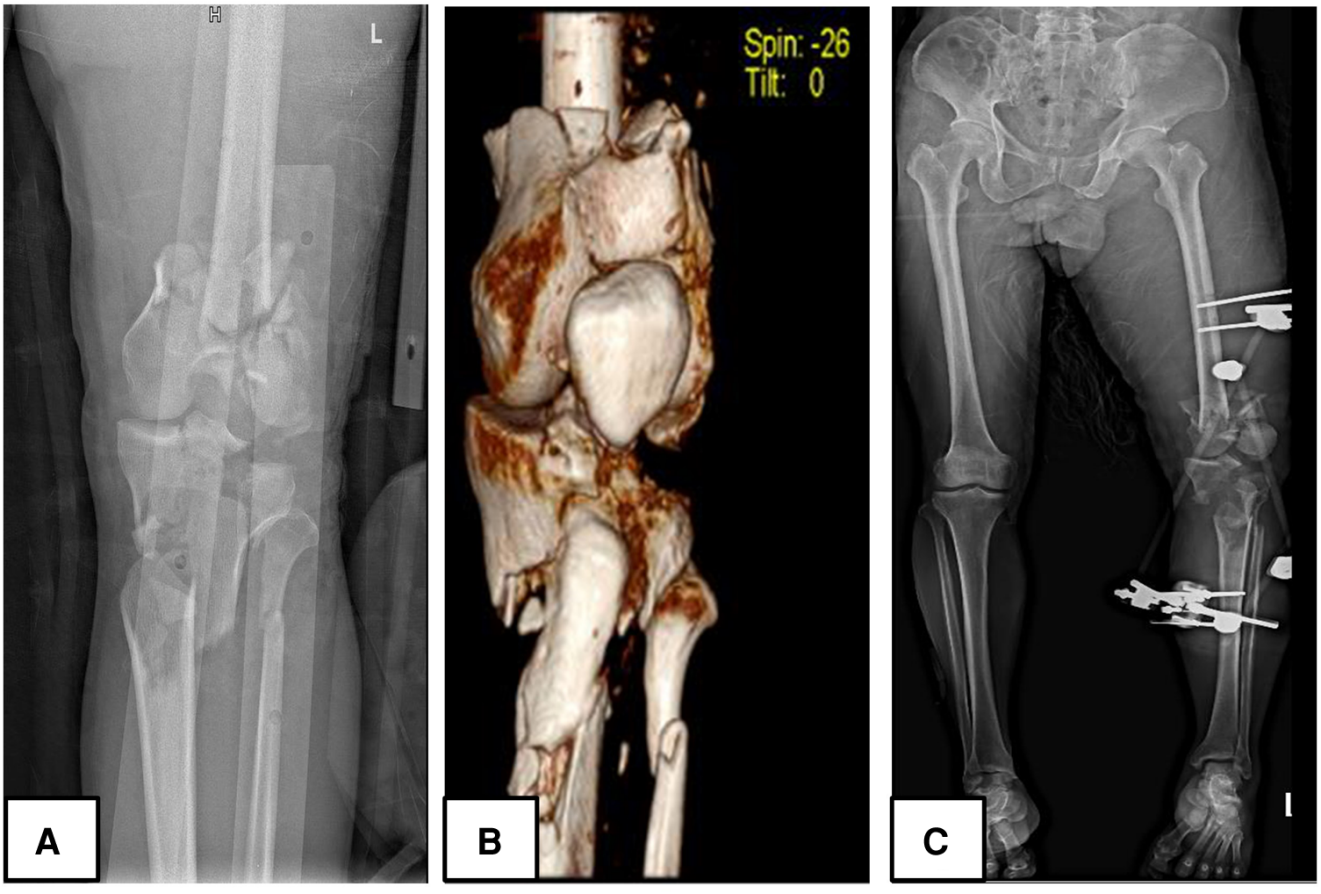
A 65 years-old male patient (patient number 34) who sustained a Fraser type IIC floating knee (left limb) following a road traffic accident managed surgically; (**A,B**) demonstrated preoperative plain radiograph and 3D reconstruction CT images respectively; (**C**) displays a postoperative plain radiograph of the lower limb, the femur and tibia were managed with knee spanning external fixation.

It goes without mention then, that in patients with Fraser type I, the antegrade intramedullary nail was the gold standard while in patients with intraarticular involvement, there was a serious need for perfect articular surface reconstruction, hence a plate was the implant of choice on the bone with the intra-articular fracture (either tibia plateau fractures in type IIa, femur intracondylar fractures in type IIb or both in type IIc). Caution was applied regarding the integrity of the articular cartilage, therefore, it was vital to avoid articular cartilage damage while managing Fraser type I fractures and in appropriate reduction of the articular surface while managing Fraser type II fractures. For this reason, there was preferred use of antegrade IM (instead of retrograde IM) in Fraser I fractures, antegrade intramedullary nails for the femur, and plating for the tibia in Fraser IIa fractures (Figure 1). Fraser IIb was often managed with a plate for femur and an intramedullary nail for tibia (Figure 2). There was a preference for plating in both tibia and femur whenever soft tissue condition was good in Type IIc. Plating was believed to achieve good reduction quality of the articular surface in Fraser II. Patients with severe soft tissue damage, who could not benefit from definitive internal fixation, were managed with a permanent external fixator (Figure 3).

In this study, the most important factors affecting postoperative clinical outcomes and complications were found to be related to articular surface involvement and the open nature of fractures. Twelve (33.33%) patients were reported to have fair to poor outcomes; of these, nine presented with fractures with articular surface involvement (Fraser IIa, IIb, and IIc), and 3 were Fraser type I. Further supporting the theory that fracture to the intraarticular surface alone was responsible for a fair to poor outcome in as much as 69.23% of patients. This hypothesis was previously supported by other authors who believed that the injury pattern including articular involvement and soft tissue damage are considered to be the most important prognostic factors (20, 24). In a study conducted by Hegazy et al. (24), three out of 15 patients who had intraarticular fractures and open 3b fractures presented poor results. Among these, there were 86.14% open injuries encountered out of which 38% of cases were grade 3b and 3c open fractures. In the current study, among all patients who presented with intraarticular fracture, only four out of 36 (11.11%) patients with intraarticular fracture and Gustilo III open fracture presented a fair outcome. In contrast, among the three cases presenting with poor clinical outcomes, two cases were not associated with open soft tissue and skin injury while 1 case was associated with Gustilo I open fracture. They however were associated with more extensive concomitant injuries, higher ISS (34–38), longer hospital LOS and worse complications; factors which influenced the management plan and the rehabilitation process.

As an illustration, in patient number 25 of our series (Table 1), the presence of bilateral lower limb compartment syndrome delayed the timing of the fracture fixation, and the presence of a contralateral tibial plateau fracture negatively affected the initiation of weight-bearing exercise which was an important component of the rehabilitation process.

Associated trauma to the head, chest, abdomen, and pelvis as well as long bones of the contralateral lower extremity was noted in around 89% of cases, highlighting the severity of this condition as well as the violent nature of the forces involved (15, 29).

Adamson et al. (29) reported 71% major associated injuries with 21% vascular injuries. Kao et al. (30) reported that from a series of 419 patients, 110 (26%) cases had a head injury, 37 (8.8%) cases had pelvis fractures, and 230 (55%) cases of patients had severe contralateral extremity injury.

Paul et al. (18) reported 6 (29%) cases of vascular injuries (1 profunda femoral artery, 1 popliteal artery, 3 posterior tibial arteries, and 1 dorsalis pedis artery) and 2 (10%) of nerve trauma in their series of 21 Patients. Hosny et al. (31) reported 4 (21.1%) vascular injuries distributed as 3 popliteal arteries and 1 posterior tibial injury. He also reported 5 (26.3%) nerve injuries involving 2 sciatic nerves and 3 common peroneal nerves. According to Bertrand et al. (14), the popliteal artery is the most commonly involved vascular structure accounting for 29% of cases. Other authors supported that, while conducting a neurovascular examination on polytrauma patients, assessment of the peripheral pulses should be mandatory; and should be accompanied by Doppler ultrasound examination together with CT angiography in selective patients (with ankle-brachial index <0.9) (9).

Thirty five out of 36 (97.22%) cases presented with concomitant injuries, in this study, out of which 14 were cases of traumatic chest injury (14 cases of rib fracture and seven cases of associated lung contusion). There were eight patients with TBI, two of whom presented with associated skull fractures. Ipsilateral patella fracture was present in nine cases.

The study reported 14 out of 35 (40%) cases of neurovascular injuries divided as follows: two (14.28%) cases of sciatic nerve injury, three (21.42%) of the tibial nerve, four (28.57%) of the common peroneal nerve, one (7.14%) superficial peroneal nerve injury, one (7.14%) femoral artery injury, three (21.42%) cases of popliteal artery injury, and one (7.14%) case of posterior tibial artery injury. Also among the 35 patients with concomitant lesions, were 17.14% cases of pelvic ring fracture, 5.71% cases of acetabular fracture, and some cases of visceral injuries. These findings were consistent with the above studies and reflected to the violence and severity of these injuries.

The literature once again supports the theory that the presence of associated injuries in floating knee patients could potentially affect the patient’s management with regards to surgical procedure delay as well as the patient’s rehabilitation (12, 32).

According to one scholar, the involvement of the knee joint and the severity of soft tissue injury on the tibia (Gustilo grade) are highly contributing factors to the risk of poor outcomes (20). According to Piétu et al. (22), in a series of 172 patients, the functional score of Karlström and Olerud revealed an excellent score in 23 patients, a good score in 38, a fair one in 35, and a bad score in 20. The contributing factors for bad functional outcome were primarily patient age, a Fraser type II lesion, a femoral fracture located at the distal third, and an open fracture.

Oh et al. (27), in a series of 18 patients, reported acceptable results in 1 patient, good results in three, and excellent results in 14 patients.

Hosny et al. (31) recently reported excellent results in two cases, good in seven, acceptable in seven, and poor in three cases after retrospectively studying 19 cases of neglected infected floating knee patients.

This study reported that from a series of 36 patients, there was an excellent score in 13, good in nine, acceptable in two, fair in nine, and poor in three patients. Among the 11 patients who presented with both fair and poor scores, four expressed extra-articular Fraser I fractures while nine had intra-articular Fraser II fractures. Four Gustilo type I, eight Gustilo II, and six Gustilo III lesions were additionally recorded. Patients with poor outcomes were found to have sustained important concomitant injuries to the contralateral limb and severe nerve injuries such as sciatic and common peroneal nerve injury. The preferred method of fracture fixation was external fixation. Factors that are all susceptible to interfere with the rehabilitation process.

One scholar reported a single case of femoral fracture non-union and two tibial fractures nonunion in 18 floating knees (27). Hosny et al. (31) in 19 cases, evaluated the postoperative distribution of complications such as malunion (in three cases), refracture (in two cases), and DVT (in four cases). All patients had pins tract infections and had been managed with close reduction and fixation using Ilizarov principles.

In this study, two cases of femoral non-union and one case of tibia non-union, were registered. The non-union was managed with reoperation, implant exchange, and bone grafting. The case of tibia non-union occurred in a patient with severe soft tissue damage (Gustilo IIIb), which was believed to be a contributing factor. The two cases of femoral non-union were not clearly understood. Additionally, there were five cases of surgical site infection, five cases of DVT, one sciatic nerve injury, and two common peroneal nerve injuries. It was still concluded that opened fracture was the most important factor for patient prognosis.

In our series, the most important complication we avoided with our protocol was death due to hypovolemic and metabolic shock syndromes.in contrast, the most common complication usually encountered was wound complication (such as infection and skin defect) and neurovascular complication; in these cases, the opinion of the microbiologist was required and the recommended antibiotics and dressing was the protocol of choice. Moreover, considering the severity of these injuries, our approach is usually multidisciplinary involving intensive care unit consultant, cardiothoracic surgeon, vascular surgeon, microbiologist, neurosurgeon, plastic surgeon and rehabilitation consultants … . All these contributed in reducing complication related from the initial injury, the treatment provided and the hospitalization. We therefore suggest that, the care of floating knee injuries must be multidisciplinary.

The current study had some limitations: First, as it is a retrospective study, there was the possibility of bias. Second, the rarity of injuries of this nature allowed the study to only be conducted on a uniquely small sample size of data. Lastly, the duration of the study and the applied inclusion criteria contributed to reducing the already small sample size.

Additionally, the evaluation of all clinical results at 12 months only could not give accurate information on other possible long-term complications. However, other forms of studies will be undertaken in regards to the floating knee injury as the number continues to grow amongst trauma patients, to strengthen the results of the current study and contribute to improving literature about the floating knee.

## 5. Conclusion

The floating knee has become an important concern in the Orthopedic and trauma field. There are several challenges in management and the topic remains controversial among trauma specialists. There is still no other study in the current literature evaluating the incidence of the floating knee among lower limb trauma patients. This study found that the incidence of this condition among lower limb trauma patients was 2.32% (0.23% per year) and that road traffic accident was the most important etiology. Articular involvement, soft tissue damage, and the presence of concomitant injuries were among the most important factors influencing the functional outcome; therefore to improve the clinical outcome, we suggest that the care of these patients should be multidisciplinary involving other specialists.

## Data Availability

The datasets presented in this study can be found in online repositories. The names of the repository/repositories and accession number(s) can be found in the article.
